# STING agonists enable antiviral cross-talk between human cells and confer protection against genital herpes in mice

**DOI:** 10.1371/journal.ppat.1006976

**Published:** 2018-04-02

**Authors:** Morten K. Skouboe, Alice Knudsen, Line S. Reinert, Cedric Boularan, Thierry Lioux, Eric Perouzel, Martin K. Thomsen, Søren R. Paludan

**Affiliations:** 1 Department of Biomedicine, Aarhus University, Denmark; 2 InvivoGen, Toulouse France; 3 Department of Clinical Medicine, Aarhus University, Denmark; Emory Vaccine Center, UNITED STATES

## Abstract

In recent years, there has been an increasing interest in immunomodulatory therapy as a means to treat various conditions, including infectious diseases. For instance, Toll-like receptor (TLR) agonists have been evaluated for treatment of genital herpes. However, although the TLR7 agonist imiquimod was shown to have antiviral activity in individual patients, no significant effects were observed in clinical trials, and the compound also exhibited significant side effects, including local inflammation. Cytosolic DNA is detected by the enzyme cyclic GMP-AMP (2’3’-cGAMP) synthase (cGAS) to stimulate antiviral pathways, mainly through induction of type I interferon (IFN)s. cGAS is activated upon DNA binding to produce the cyclic dinucleotide (CDN) 2’3’-cGAMP, which in turn binds and activates the adaptor protein Stimulator of interferon genes (STING), thus triggering type I IFN expression. In contrast to TLRs, STING is expressed broadly, including in epithelial cells. Here we report that natural and non-natural STING agonists strongly induce type I IFNs in human cells and in mice *in vivo*, without stimulating significant inflammatory gene expression. Systemic treatment with 2’3’-cGAMP reduced genital herpes simplex virus (HSV) 2 replication and improved the clinical outcome of infection. More importantly, local application of CDNs at the genital epithelial surface gave rise to local IFN activity, but only limited systemic responses, and this treatment conferred total protection against disease in both immunocompetent and immunocompromised mice. In direct comparison between CDNs and TLR agonists, only CDNs acted directly on epithelial cells, hence allowing a more rapid and IFN-focused immune response in the vaginal epithelium. Thus, specific activation of the STING pathway in the vagina evokes induction of the IFN system but limited inflammatory responses to allow control of HSV2 infections *in vivo*.

## Author summary

Herpes simplex virus (HSV)-2 is the leading cause of genital ulcers, and HSV-2 infection has also been reported to amplify HIV-transmission. So far, all attempts at making an effective anti-HSV2 vaccine have failed. In recent years, there has been an increasing interest in immunomodulatory therapy as a means to treat infections. Although the TLR7 agonist imiquimod has been shown to have antiviral activity in individual patients, no significant effects were observed in clinical trials, and the compound also exhibited significant side effects including local inflammation. Type I interferon (IFN)s are key players in antiviral defense, and it is now known that the DNA sensor cyclic GMP-AMP synthase produces the cyclic di-nucleotide (CDN) 2’3’-cyclic GMP-AMP (cGAMP), which activates the adaptor protein STING to induce IFN expression. In this work we show that natural and non-natural CDNs activate strong type I IFN responses *in vivo* without stimulating significant expression of genes driven by the transcription factor NF-κB, which induces inflammation. Application of CDNs at epithelial surfaces gave rise to local IFN activity, but only limited systemic responses. Importantly, all tested treatment regimens, strongly reduced replication of HSV-2 in a model for genital herpes, and significantly reduced development of disease. Finally, when comparing to TLR agonists, CDNs showed the best profile with strong IFN response specifically in the epithelial cells and limited induction of inflammation.

## Introduction

Virus infections may cause acute and chronical diseases, and there is therefore a need for development of efficient treatments. Significant improvements have been made in the development of therapeutics that target specific viral molecules, such as the HIV reverse transcriptase and the hepatitis C virus NS5A protein [1, 2]. Despite this, satisfactory treatments are not available for many virus infections, and there is also a need for treatments acting in a broader manner, and which are less sensitive to viral development of resistance. Herpes simplex virus (HSV)-2 is the leading cause of genital ulcers [3, 4] with an estimated 500 million infected people globally [5]. Although intensively pursued, all attempts at making an effective anti-HSV2 vaccine have failed [4]. The current standard treatment is acyclovir or derivatives, which target the viral thymidine kinase [6], and although generally efficient if treatment is initiated early, there are reports of development of resistance in immunosuppressed patients receiving long-term treatment [6]. The need for new and better anti-HSV2 treatments is underpinned by several factors, including the ability of this virus to cause neonatal herpes [3], the role of HSV2 in amplifying HIV-transmission, which has been reported to account for up to half of all new transmissions in areas of high HSV2 seroprevalence [7, 8], and the recently reported association to increased rates of autism-spectrum disorders [9].

In addition to directly targeting the virus, antiviral treatments can stimulate host immune responses. Previously tested experimental immune modulatory therapies for virus infections have mainly focused on agonists for Toll-like receptors (TLRs). The TLR7-agonist imiquimod and the mixed TLR7/8-agonist resiquimod induce interferon (IFN)α, and imiquimod is the first approved topically active TLR7 agonist used to treat human papilloma virus (HPV), but has failed to show significant efficacy against HSV2 infection [10–12], although cases have been reported with benefit of imiquimod 5% cream for treatment of herpes labialis and genital herpes [13, 14]. Furthermore, pretreatment of mice with oligodeoxynucleotide TLR9 agonists has been shown to lower the viral load in the brain in an HSV-1 encephalitis model [15]. Common to the aforementioned TLRs is their role in innate recognition of foreign nucleic acids in the endosomal compartment [16]. However, TLRs are mainly expressed in leukocytes, and to a much lesser extent in epithelial cells [17]. Another protein involved in nucleotide sensing is the cyclic GMP-AMP synthase (cGAS), which is localized in the cytoplasm, and hence is a sensor of mislocalized endogenous or exogenous DNA [18]. We and others have shown that cGAS plays an intrinsic role in mounting protective immune responses against DNA viruses, including HSV-1 [19–21]. When cGAS senses dsDNA in the cytosol, it produces the second messenger 2’3’-cyclic GMP-AMP (2’3’-cGAMP) which activates the adaptor protein Stimulator of IFN-genes (STING) on the ER [18, 22, 23]. STING dimerizes and traffics to the ER-Golgi intermediate compartment (ERGIC) where it recruits the TANK-binding kinase 1 (TBK1), which in turn phosphorylates IFN-regulatory factor 3 (IRF3) that translocates as a dimer to the nucleus where it initiates transcription of type I IFN genes [24]. Type I IFNs are secreted cytokines, which work in auto- and paracrine manners via the IFNα receptor (IFNAR) to upregulate IFN-stimulated genes (ISGs) that target specific steps in the viral life cycle to inhibit replication [24].

In addition to the action of 2’3’-cGAMP inside the DNA-stimulated cell, this CDN is also able to exert effects inside other cells through at least two distinct mechanisms, either juxtacrinely by diffusing through gap junctions [25] or endocrinely by being packaged into newly forming virions [26, 27]. Serum contains the ectonucleotide pyrophosphatase/phosphodiesterase (ENPP)1, which metabolizes 2’3’-cGAMP [28], thus hindering extensive circulation and endocrine stimulation by cell-free 2’3’-cGAMP. However, free 2’3’-cGAMP (and other cyclic dinucleotides (CDNs)) has the capacity to pass the cell membrane and activate STING [29]. Therefore, CDNs could potentially stimulate immune responses locally and over longer distances.

The use of STING agonists for treatment of disease has been tested in a series of models. The small molecule DMXAA (vadimezan) was shown to have anti-tumor effects in a mouse model [30] before its mechanism of action as a STING ligand was discovered [31], and it was tested in a clinical phase III efficacy trial of treatment of advanced non-small-cell lung cancer [32]. It was subsequently shown that DMXAA does not stimulate human STING, as opposed to murine STING, due to a single amino acid difference between human and murine STING at a residue mediating interaction between murine STING and DMXAA [31, 33]. Another interesting difference between STING in humans and mice is that human STING is activated more potently by 2’3’-linked CDNs (produced in metazoan cells) than 3’3’-linked CDNs (produced in non-metazoan cells), while murine STING is activated to comparable degrees by the two types of CDNs [29]. In the past few years, STING ligands have been investigated for anti-tumor activity in a variety of mouse models [34, 35], anti-inflammatory effects in an experimental autoimmune encephalitis mouse model [36], as well as for potential as adjuvants in vaccines [37–41]. However, whether STING agonists could have antiviral therapeutic effects has remained under-explored. In this study, we investigate the antiviral action of natural and non-natural CDNs in a murine model of genital HSV2 infection. We demonstrate that different CDNs stimulate the IFN pathway to varying degrees, and that intraperitoneal (i.p.) and local delivery stimulates rapid IFN response with associated antiviral function. Lastly, we show that local application of a CDN to the vaginal mucosa confers full protection against genital HSV2 infection in both wild type and immunodeficient *cGas*^-/-^ mice. The effects of CDN treatment are superior to TLR7 and 9 agonists, based on high antiviral activity, IFN-biased response with low TNF expression, and targeted stimulation of epithelial cells.

## Results

### STING agonists differentially stimulate human cell types, which enables cross-talk-mediated protection against HSV2 infection

**Fig 1:**
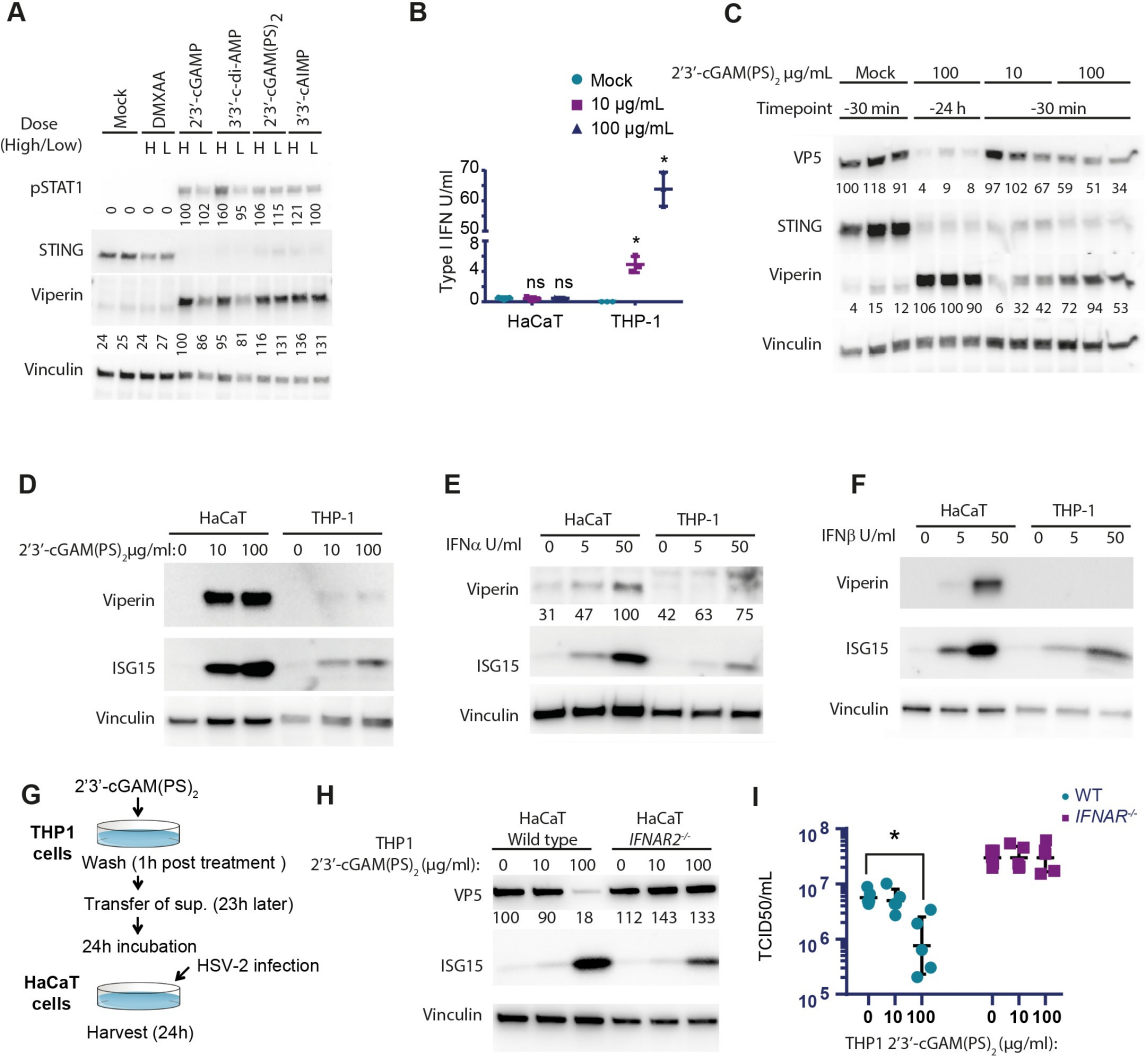
STING agonists induces type I IFN and anti-viral effect in human cells. (**A**) HaCaT cells were permeabilized with digitonin before stimulated with different STING agonists at a low (L: 10μg/ml) or high (H: 100μg/ml) concentration for 24 hours. Cell lysates were used for Western blotting for pSTAT1, STING and viperin. (**B**) HaCaT and THP-1 cells were stimulated with 2’3’-cGAM(PS)_2_ for 24 hours and type I IFN activity was measured in the supernatant (n = 3, * = p<0,05). (**C**) HaCaT cells were permeabilized with digitonin and treated with 2’3’-cGAM(PS)_2_ before or after infection with HSV2 (MOI 0.1) for 24 hours. Cell lysates were used for Western blotting for VP5, STING and viperin (n = 3). (**D, E, F**) HaCaT and THP-1 cells were stimulated with 2’3’-cGAM(PS)_2_, IFNα or IFNβ for 24 hours and levels of viperin and ISG15 were determined by Western blotting. (**G, H, I**) THP-1 cells were stimulated with 2’3’-cGAM(PS)_2_ for 24 hours before the media were transferred to wild type or *IFNAR2*^*-/-*^ HaCaT cells. HaCaT cells were infected 24 hours later with HSV2 (MOI 0.1) for 24 hours. Cell lysates were used for Western blotting for VP5 and ISG15 and the media were used to determine the HSV-2 virus load. For all Western blots, Vinculin was used as loading control. n = 3, a representative sample is showed. NT, non-treated. Statistics, (**B, I**) Two-way ANOVA with Šidák’s multiple comparisons test; p(interaction)<0.05.

To test whether STING agonists can induce antiviral response against HSV2, we treated human keratinocytes (HaCaT) with five different STING agonists. Keratinocytes are specialized epithelial cells and are the primary cells involved in clinical lesions caused by HSV2. The STING agonists tested were DMXAA (5,6-dimethylxanthenone-4-acetic acid, vadimezan), 2’3’-cGAMP; 3’3’-cyclic di-adenosine monophosphate (3’3’-c-di-AMP), and two cyclic dinucleotide analogs, namely the Rp,Sp isomer of the bisphosphorothioate analog of 2’3’-cGAMP (2’3’-cGAM(PS)_2_ (Rp/Sp)), and 3’3’-cyclic adenosine monophosphate- inosine monophosphate (3’3’-cAIMP). As expected, DMXAA did not induce expression of the ISGs viperin and ISG15 or phosphorylation of STAT1 (Fig 1A), as measured 6 h after stimulation. For the CDNs, we observed phosphorylation of STAT1, upregulation of viperin, and lower levels of STING, indicating its activation and subsequent degradation (Fig 1A). Despite the observed phosphorylation of STAT1 and induction of viperin, we did not find detectable levels of type I IFN in the supernatants of HaCaT cells stimulated with 2’3’-cGAM(PS)_2_ (Rp/Sp) (Fig 1B). In sharp contrast to HaCaT cells, the human monocyte-like cell line THP-1 responded to 2’3’-cGAM(PS)_2_ (Rp/Sp) stimulation with a very strong induction of type I IFN production, but limited induction of ISGs, and was also less responsive to IFNα or -β treatment (Fig 1B, 1D–1F). Since HaCaT cells were highly responsive to IFNα or -β treatment (Fig 1E and 1F), we cannot exclude that these cells did induce type I IFN levels below the detection limit, which stimulated significant IFN signaling and gene expression. Of note, for the data shown in Fig 1B, 1D and 1H, cells were not permeabilized before treatment with 2’3’-cGAM(PS)_2_ (Rp/Sp), thus suggesting direct cellular entry without additional delivery systems. Collectively, the data suggest that monocytes produce much higher levels of type I IFN in response to STING agonists than keratinocytes, which however are more responsive to IFN stimulation than monocytes.

To investigate whether the CDN stimulation could protect against an HSV2 infection, we treated HaCaT cells with 2’3’-cGAM(PS)_2_ (Rp/Sp) either 24 h or 30 min before infection. In cells treated with CDN 24 h prior to infection, we observed strongly reduced levels of the major capsid protein, viral protein 5 (VP5) (Fig 1C), indicating a protection of the cells from infection. The reduced accumulation of viral proteins was also seen in cells treated with high dose of 2’3’-cGAM(PS)_2_ (Rp/Sp) 30 min before infection. Since, THP-1 cells produced high levels of type I IFN upon CDN stimulation, we wanted to test whether this could contribute to antiviral activity in a setting with crosstalk between different cell types. Therefore, supernatants from 2’3’-cGAM(PS)_2_ (Rp/Sp) -treated THP-1 cells were transferred to WT and *IFNAR2*^*-/-*^ HaCaT cells, which were subsequently infected with HSV2 (Fig 1G). Interestingly, treatment with the supernatants from stimulated THP1 cells led to a strong reduction of HSV2 replication in WT, but not *IFNAR2*^*-/-*^ HaCaT cells (Fig 1H and 1I). Collectively, these data suggest that CDNs differentially stimulate keratinocytes and monocytes, and that crosstalk between the cell-types enables the full antiviral response, which is dependent on type I IFNs.

### Systemic delivery of CDNs stimulates IFN responses in several tissues in mice

**Fig 2:**
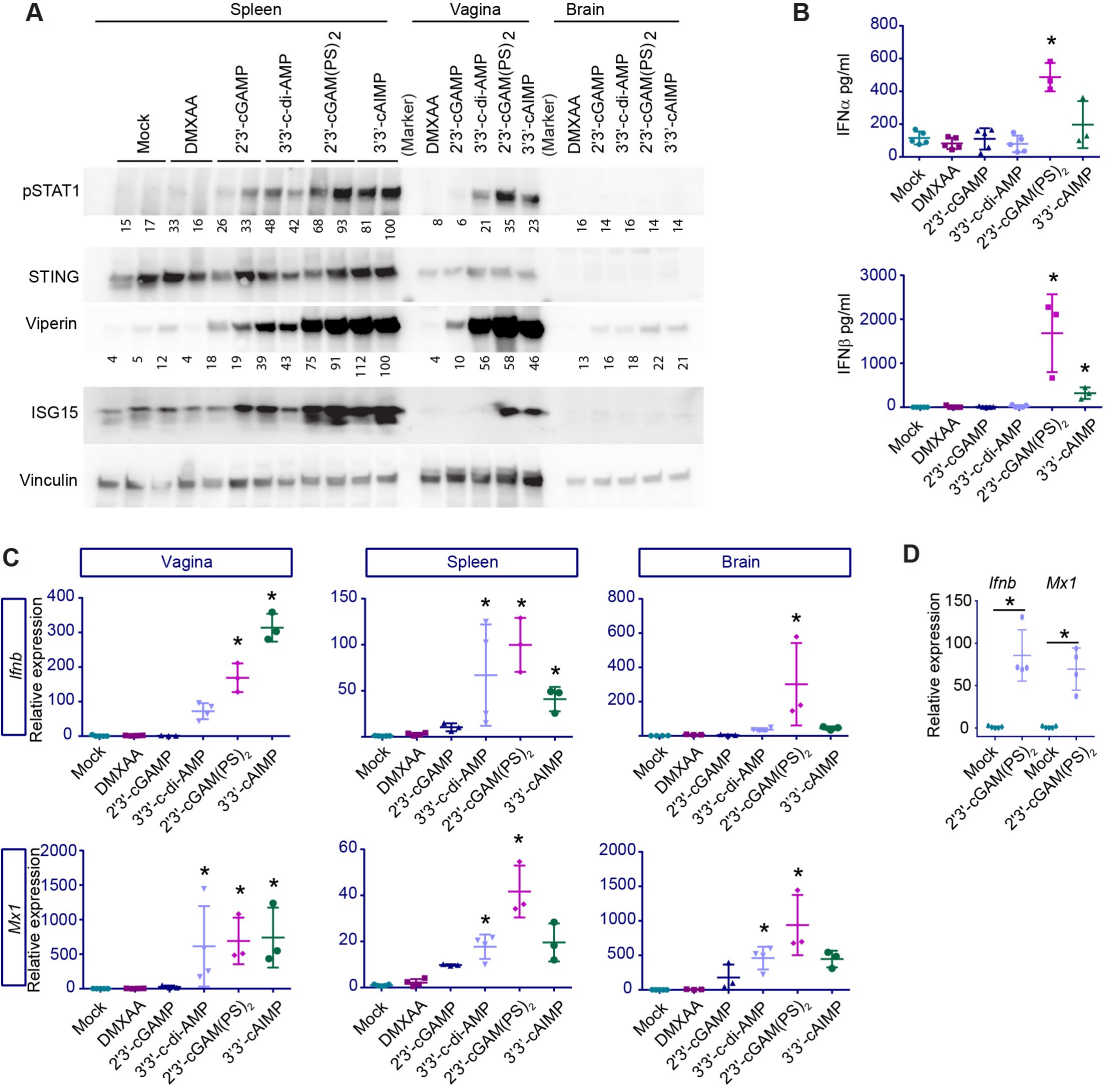
STING agonists induces type I IFN expressing *in vivo*. Equimolar (1.687x10^-7^ mol) doses of STING agonists 2’3’-cGAMP (121 μg/mouse), 3’3’c-diAMP (119 μg/mouse), 2’3’-cGAM(PS)_2_ (125 μg/mouse), 3’3’-cAIMP (111 μg/mouse) or DMXAA (95 μg/mouse, STING activation by DMXAA requires two molecules (3.374x10^-7^ mol)) were administrated to mice i.p. Samples were collected 6 hours later for further analysis. (**A**) Levels of phosphorylated STAT1 (pSTAT1), STING, viperin and ISG15 were determined by Western blotting of spleen, vagina and brain tissues. Vinculin was used as loading control. n = 5, two representative samples are shown. (**B**) IFNα and IFNβ in the serum determined by ELISA. n = 3–5. * = p<0.05 compared to mock. (**C**) Expression for *Ifnb* and *Mx1* mRNA in tissues samples from vagina, spleen and brain, normalized to GAPDH. n = 3–5. * = p<0,05 compared to mock. **(D**) Mice were perfused prior to isolation of brain samples and gene expressions of *Ifnb* and *Mx1* mRNA were measured. n = 3–5. * = p<0,05 compared to mock. Statistics, (**B-D**) Kruskal-Wallis test with Dunn’s multiple comparisons test.

To determine the effect and potency of STING agonists in animals, we injected the compounds i.p. into wild type (WT) mice in equimolar doses (Fig 2). At 6 h post treatment, we observed phosphorylation of STAT1 and upregulation of viperin and ISG15 (Fig 2A). This was most pronounced in the mice treated with the non-endogenous CDNs, and to a much lesser extent with 2’3’-cGAMP. Very strong responses were observed in serum, spleen, and the vagina (epithelial surface), whereas systemic treatment with the compounds gave rise to very low responses in the brain (Fig 2A–2C). The lack of a response to DMXAA is explained by the low dose used in this setting, as we found that DMXAA potently stimulated an IFN response when the dose was increased (S1A Fig). Further analysis of the induction of expression of IFNs and ISGs by systemically delivered STING agonists confirmed that the non-endogenous CDNs potently stimulated the IFN pathway in the vagina and the spleen, and that DMXAA and 2’3’cGAMP were much less potent *in vivo* (Fig 2C). We also found that CDNs stimulated modest induction of the nuclear factor (NF)κB-induced genes *A20*, *Il6*, *and Tnfa* (S1B Fig), thus suggesting that direct activation of the STING pathway in mice does not lead to strong activation of the NFκB pathway. Furthermore, mice deficient for STING did not respond to 2’3’-cGAM(PS)_2_ (Rp/Sp) treatment, underlining that STING is the target for the molecules tested (S1C Fig). However, since an NFκB responsive reporter gene was readily activated by STING agonists in THP1 cells (S1D Fig), we are reluctant to conclude that NFκB is not involved in the gene induction program induced upon STING activation.

To ensure that the small, but significant, 2’3’-cGAM(PS)_2_ (Rp/Sp)-induced IFN response we observed in the CNS was not derived from blood left over in the vasculature following harvest of brain tissue, we injected WT mice i.p. with 2’3’-cGAM(PS)_2_ (Rp/Sp) and performed a whole-animal transcardial perfusion of PBS to remove any residual blood from the peripheral vasculature before taking out the brain of the animals. Following this procedure, we still found a significant upregulation of the expression of *Ifnb* and *Mx1* in the brain (Fig 2D). This indicates that the IFN response in the brain tissue originates from resident cells, suggesting that among the CDNs tested, 2’3’-cGAM(PS)_2_ (Rp/Sp) may cross the blood-brain-barrier and exert an effect directly in the CNS. Taken together, these data suggest that systemic administration of non-endogenously produced CDNs yields an IFN response in several different tissues, including lymphoid organs, epithelial surfaces, and the CNS.

### STING agonists provoke an ISG-response in the vaginal mucosa

**Fig 3:**
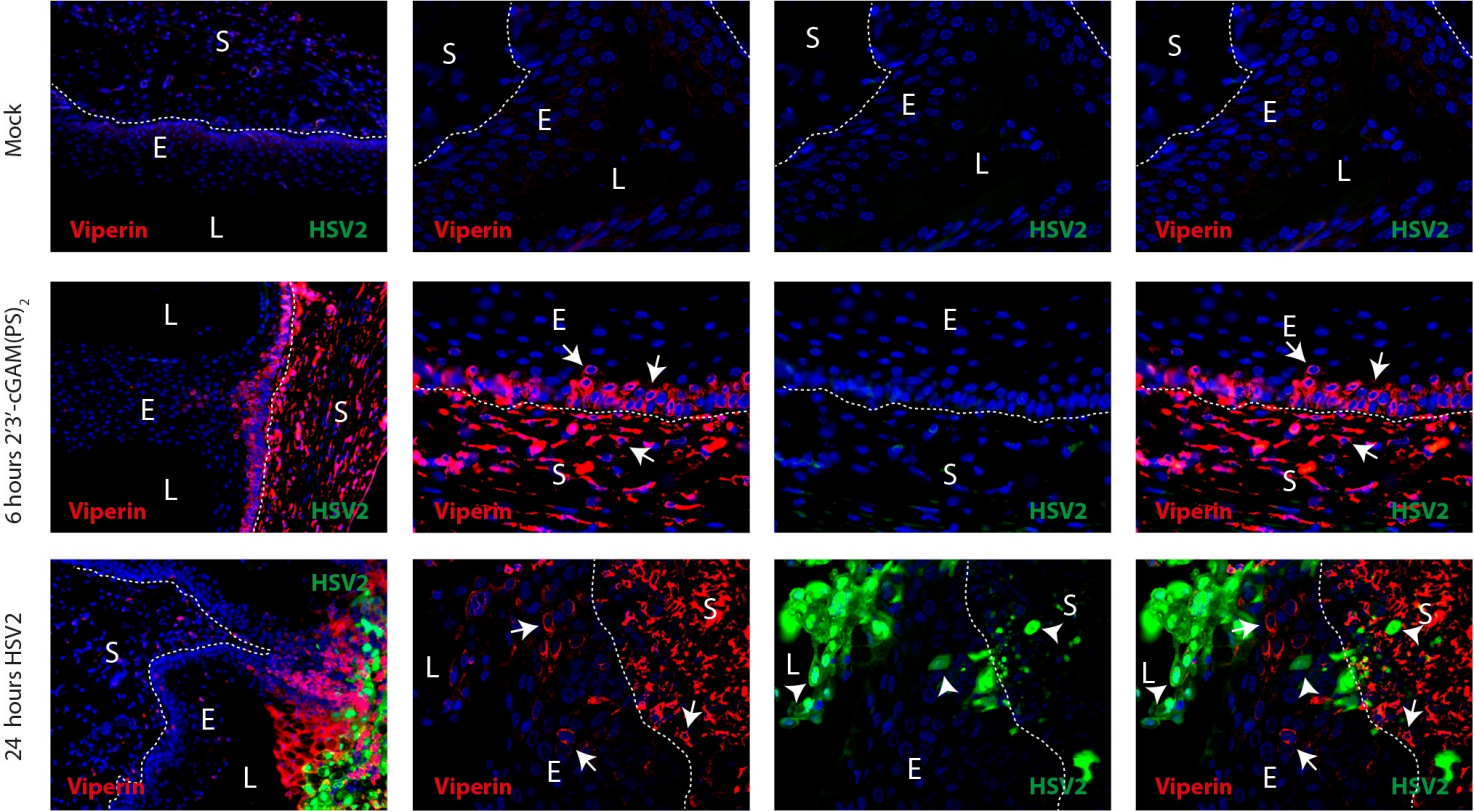
HSV2 and STING agonists induce antiviral genes in vaginal epithelium. Mice were injected i.p. with 2’3’-cGAM(PS)_2_ (125 μg/mouse) or infected with HSV2 (6.7×10^4^ p.f.u.). Tissues were isolated from mice 6 h and 24 h after CDN stimulation and HSV2 infection, respectively. Paraffin sections of the vaginal tissues were stained for viperin (red) and HSV2 (green). DAPI (blue) marks the nuclei and the dotted white lines mark basal membrane between the epithelium and stroma. White arrows highlight examples of viperin positive cells, and arrowheads mark examples of HSV2-infected cells. L = lumen, E = epithelium, S = stroma. n = 3. One representative picture is shown for each staining and treatment group.

To investigate how the STING agonists affected the IFN response in the tissue, we chose to look more closely at the vagina, which is one of the main portals of entry for viral infections. Using immunohistochemical staining of vaginal tissues, we found that systemic delivery of 2’3’-cGAM(PS)_2_ (Rp/Sp) induced strong expression of the ISG viperin by cells in the stroma (Fig 3). Furthermore, epithelial cells located close to the basal membrane were positive for viperin, while epithelial cells located to the luminal side had less expression. Vaginal tissue from mice infected locally with HSV2 showed strong viperin expression in cells located to the area of infection. Few cells co-expressed viperin and HSV2 antigens, but cells around the HSV2-infected cells expressed high levels of viperin. This included a range of cell types, including epithelial, stroma, and potentially also immune cells.

### Systemic administration of STING agonists improves the survival of mice infected with HSV2

**Fig 4:**
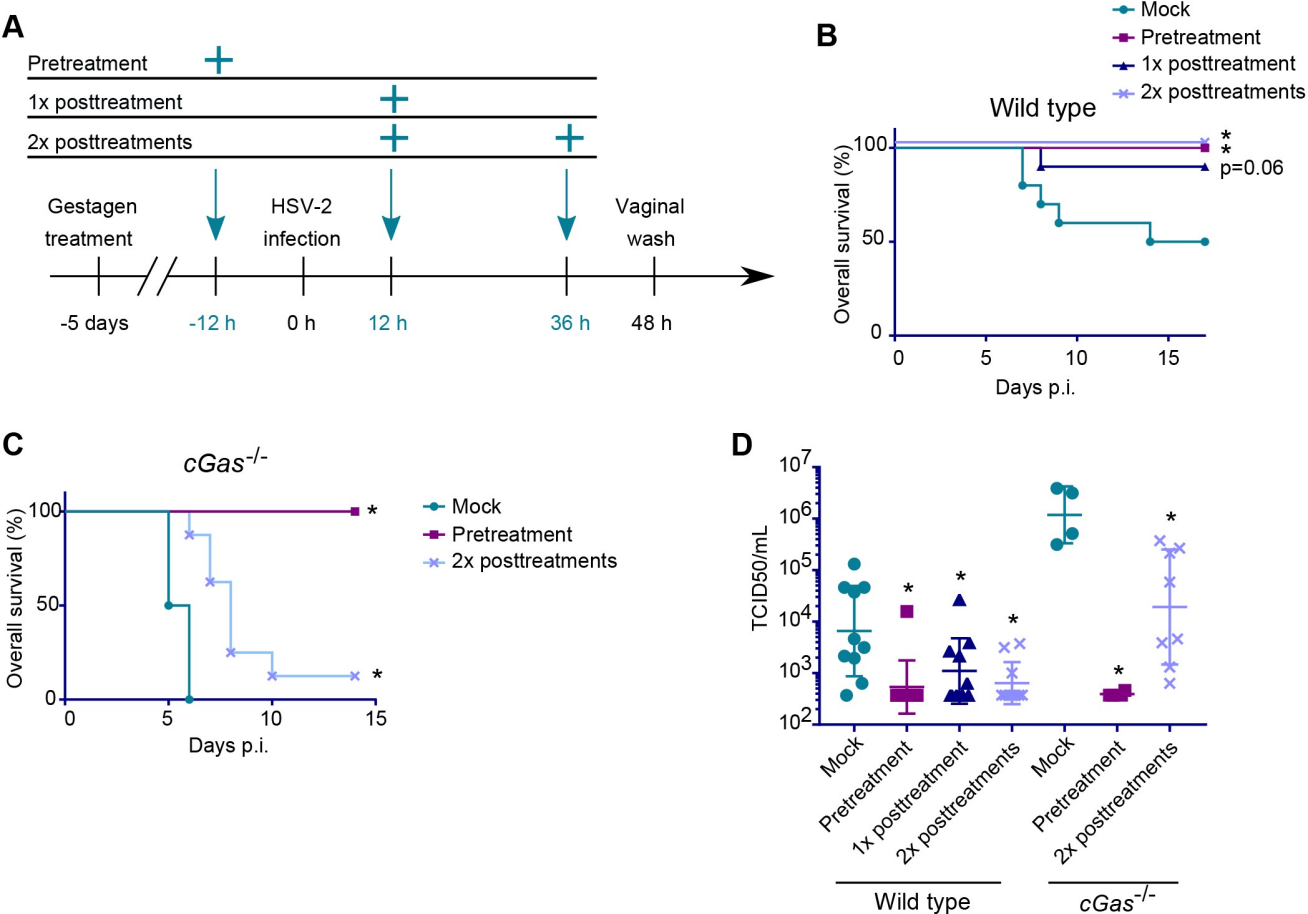
Systemic treatment with STING agonists confers protection against genital HSV2 infection. Wildtype and *cGas*^-/-^ mice were treated with 2’3’-cGAM(PS)_2_ (Rp/Sp) (125 μg/mouse) and infected intravaginally with HSV2 (6.7×10^4^ p.f.u.). (**A**) Illustration of the timeline for the treatment regimens. (**B, C**) Overall survival for HSV2-infected and treated wildtype (**B**) or *cGas*^*-*/-^ (**C**) mice. n = 6–10. * = p<0,05 compared to mock. **(D**) HSV2 titer (TCID50) in vaginal washes collected 48 hours post infection. n = 6–10. * = p<0,05 compared to mock in the same genotype. Statistics, (**B**, **C**) Log-rank test with Holm-Bonferroni correction. (**D**) One-way ANOVA of log_10_-transformed data with Dunnett’s multiple comparisons test.

To explore whether the upregulation of ISGs in the vaginal mucosa could play a role in control of infection *in vivo*, we set up three different treatment regimens with i.p. injections of 2’3’-cGAM(PS)_2_ (Rp/Sp) before or after HSV2 infection. The regimens were (CDN treatment relative to infection): one pretreatment, one post-treatment, or two post-treatments (Fig 4A). When treating WT mice, the CDN pretreatment and two-time post-treatments showed significantly improved survival as compared to non-treated controls, and the one-time post-treatment group suggested improved survival, but the data did not reach statistical significance (p = 0.06) (Fig 4B). We also tested *cGas*^*-/-*^ mice, since they cannot produce 2’3’-cGAMP upon immune recognition of HSV2, but can be stimulated by external delivery of STING agonists. When treating *cGas*^*-/-*^ mice, however, only the pretreated group had a 100% overall survival, while almost all the post-treated mice eventually succumbed to the infection, although the two-times post-treatment led to significantly improved survival, (Fig 4C). The differences in the effects of two-time post treatment on survival between WT and *cGas*^*-/-*^ mice may be explained by the role of endogenous activation of STING signaling upon genital HSV2 infection, as reported previously [42]. All groups receiving CDN treatment, regardless of the genotype, had significantly lower viral loads in the vagina when compared to their respective controls (Fig 4D). Taken together, these data demonstrate that activation of the STING pathway with the non-endogenous CDN 2’3’-cGAM(PS)_2_ (Rp/Sp) mounts a potent antiviral defense, the efficacy of which depends on the timing and repetition of treatment as well as the immunocompetence of the mouse.

### Local administration of STING agonists yields a robust local and a weaker systemic IFN response which confers protection against HSV2 infection

**Fig 5:**
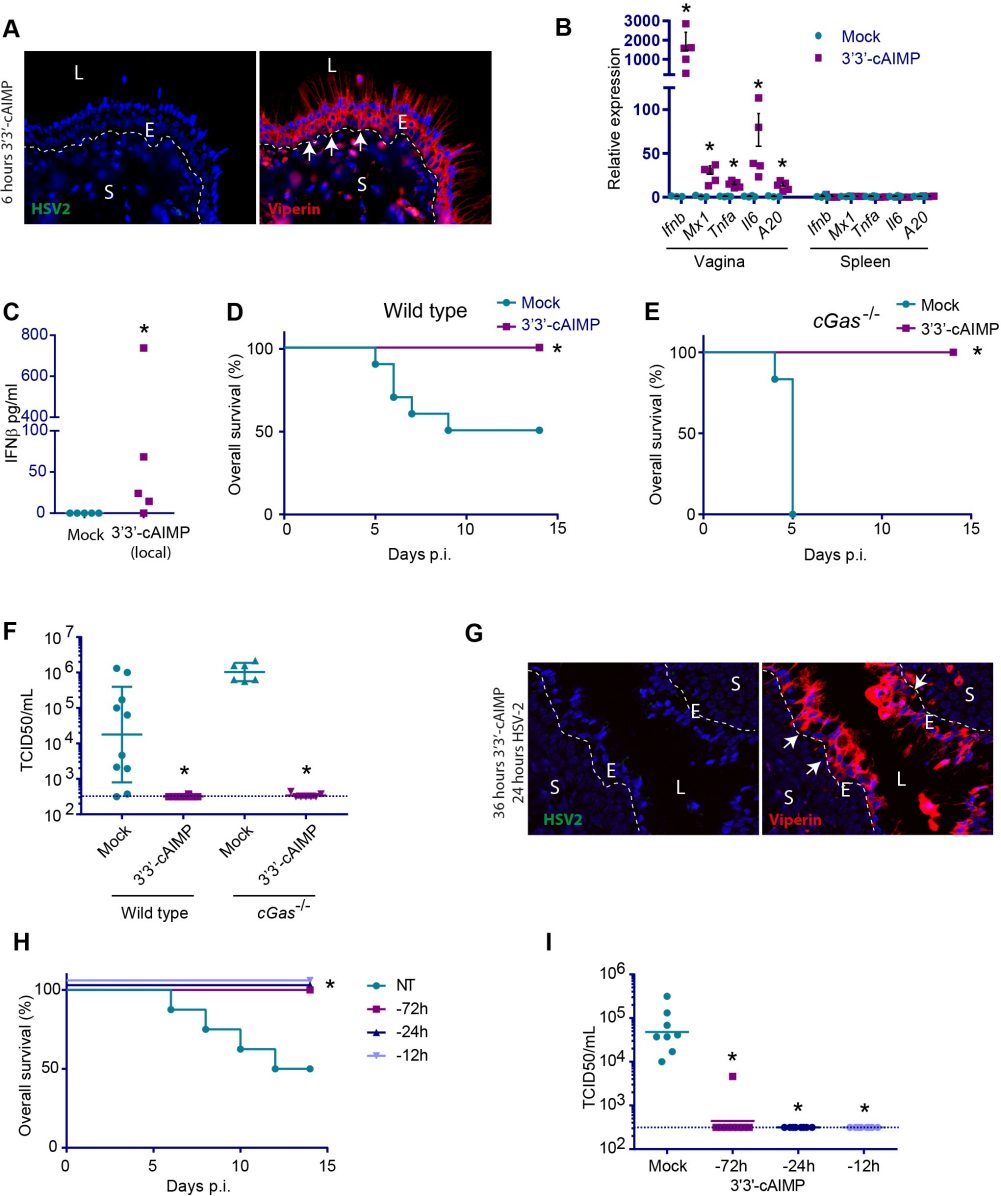
Local application of STING agonists protects against genital HSV2 infection. Mice were anesthetized for 30 min and 250 μg 3’3’-cAIMP was applied to the vagina. (**A**) Tissues were isolated from mice 6 h after CDN stimulation. Paraffin sections of the vaginal tissues were stained for viperin (red) and HSV2 (green). DAPI (blue) marks the nuclei and the dotted white lines mark basal membrane between the epithelium and stroma. White arrows highlight examples of viperin positive cells. L = lumen, E = epithelium, S = stroma. n = 4. One representative picture is shown for each staining. (**B**) After 6 hours of 3’3’-cAIMP treatment (no infection), gene expression was measured in vaginal and spleen samples. The expression levels were normalized to GAPDH. n = 3–5. * = p<0.05. (**C**) IFNβ levels. in serum from mice treated with 3’3’-cAIMP for 6 hours. n = 5. (**D, E**) Overall survival for wildtype (**D**) or *cGas*^-/-^ (**E**) mice treated with 3’3’-cAIMP and infected with HSV2 12 h later. n = 6–10. * = p<0,05 compared to mock. (**F)** HSV2 titer (TCID50) in vaginal washes (48 hours p.i.) from wildtype or *cGas*^-/-^mice treated with 3’3’-cAIMP and infected with HSV2 12 h later. n = 6–10. * = p<0,05 compared to mock treated in the same genotype. (**G**) Tissues were isolated from mice treated intravaginally for 12 h with 3’3’-cAIMP followed by 24 h infection with HSV-2. The samples were prepared and analyzed as in A. (**H)** Overall survival and (**I**) virus load for wildtype mice pre-treated with 3’3’-cAIMP 12, 24 or 72 h prior to virus infection. n = 8. * = p<0,05 compared to mock treated group. Statistics, (**B**) Kruskal-Wallis test with Dunn’s multiple comparisons test. (**C**, **F, I**) Mann-Whitney-Wilcoxon U test. (**D, E, H**) Log-rank test.

Given the clear antiviral action of CDNs in the vagina together with the systemic immune activation after i.p. treatment, we wanted to evaluate whether direct administration of CDNs to epithelial surfaces would also stimulate a protective response and with less systemic effects. For these experiments, we used 3’3’-cAIMP, which showed *in vivo* responses very similar to 2’3’-cGAMP (PS)_2_ (Rp/Sp) and has a high solubility, thus allowing 250 μg to be applied in 20 μl saline. Six hours after application of 3’3’-cAIMP to the vaginal mucosal surface, we observed very strong staining for viperin throughout the epithelial layer (Fig 5A). Interestingly, this was in contrast to mice receiving systemic CDN treatment, where viperin staining was strongest in cells localizing to the stroma and inner epithelium (Fig 3). In vaginal tissue from the mice receiving local CDN treatment, a strong upregulation of both *Ifnb* was observed and modest induction of *Tnfa*, *Il6*, and *A20*, (Fig 5B). In contrast to this, no IFN signature was observed in the spleen, which has a very high expression of STING (Fig 2A). However, we did detect elevated IFNβ protein levels in serum from some, but not all the mice treated with 3’3’-cAIMP in the vagina (Fig 5C). To examine the antiviral response stimulated by locally administered CDN, WT or *cGas*^*-/-*^ mice were treated in the vagina with 3’3’-cAIMP and infected with HSV2. Following this treatment, both genotypes showed complete protection against disease, and no detectable virus in the vaginal lavage (Fig 5D–5F). Staining of tissue sections from 3’3’cAIMP-treated HSV2-infected mice failed to reveal virus-infected cells, which was readily seen in the absence of CDN treatment (Figs 5G and 3). Finally, we examined how long the CDN treatment could be separated from HSV2 infection temporally. Local treatment with 3’3’-cAIMP up to 72 h prior to HSV2 infection conferred full protection against genital herpes and suppression of viral replication (Fig 5H and 5I). Taken together, these data suggest that local administration of a STING agonist to a mucosal surface confers local protection against viral infections with only mild systemic activation of the IFN system.

### STING agonists induce IFN responses in the vaginal epithelium faster and more efficiently than TLR agonists

**Fig 6:**
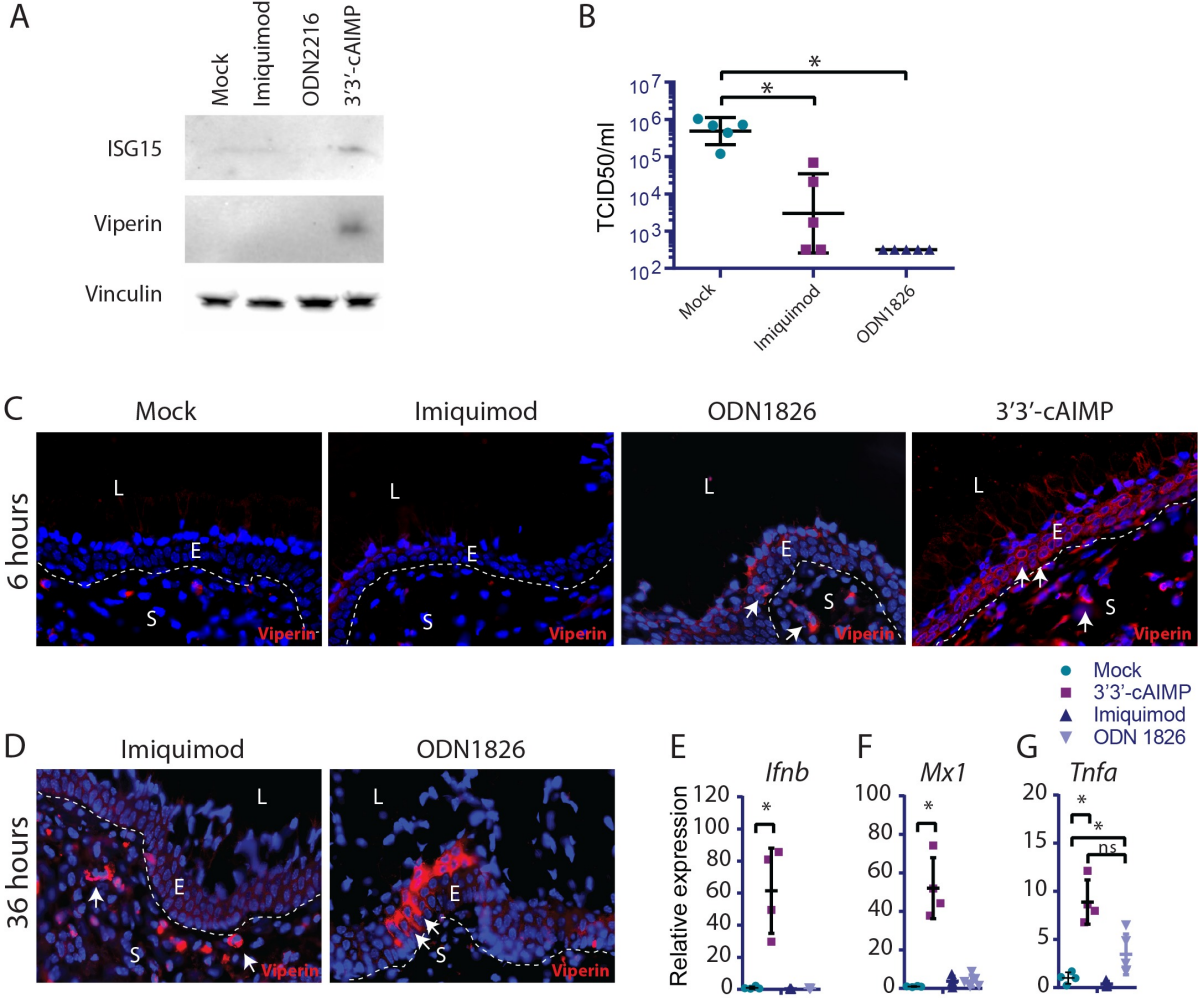
STING agonists induce IFN responses in the vaginal epithelium faster and more efficiently than TLR agonists. (**A**) HaCaT cells were treated with imiquimod (1μg/ ml), ODN2216 (1μg/ ml), and 3’3’-cAIMP (100μg/ ml) for 24 h. Levels of ISG15 and viperin were determined in the cell lysate by Western blotting. (**B**) Mice were treated intravaginally with imiquimod or ODN1826 (25 μg per mouse) 12 h prior to infection with HSV2. Vaginal washes were collected 48 h p.i. and viral load was determined. n = 5 per group. (**C-D**) Mice were anesthetized for 30 min and imiquimod, ODN1826, or 3’3’-cAIMP was applied to the vagina. Tissues were isolated (**C**) 6 h or (**D**) 36 h after treatment. Paraffin sections of the vaginal tissues were stained for viperin (red). DAPI (blue) marks the nuclei and the dotted white lines mark basal membrane between the epithelium and stroma. White arrows highlight examples of viperin positive cells. L = lumen, E = epithelium, S = stroma. n = 4. One representative picture is shown for each staining. (**E-G**) RNA was isolated from vaginal tissue treated as indicated for 6 h, and levels of *Ifnb*, *Mx1*, and *Tnfa* mRNA were determined by RT-qPCR. n = 4–5. mRNA levels were normalized to *Gapdh* and shown as relative levels of expression compared to mock-treated mice. (**B, E-G**) Statistics, Kruskal-Wallis test with Dunn’s multiple comparisons test. * = p<0,05 compared to mock treated.

Previous attempts to take advantage of innate immune activation to control genital herpes infections, showed promising results in mice, but only limited clinical effects were obtained, and clear inflammatory side effects were observed [10, 11]. TLR7 and 9 are mainly expressed on leukocytes, notably plasmacytoid dendritic cells, while HSV2 replicates in the vaginal epithelium. Therefore, we were interested in directly comparing induction of ISG expression by STING agonists relative to TLR agonists. We first treated HaCaT cells with imiquimod, ODN2216 or 3’3’-cAIMP and examined for expression of ISGs. While none of the TLR agonists induced ISG expression, this was observed following CDN treatment (Fig 6A). Next, we were interested in examining how the different agonists affected induction of the IFN effector proteins, the ISGs, in the vaginal epithelium. First, we observed in agreement with previous results that ODN1826, similarly to 3’3’-cAIMP, totally blocked genital HSV2 replication in mice, while imiquimod has a partial effect [43, 44] (Fig 6B). Interestingly, when vaginal tissue sections from mice treated locally with the TLR agonists for 6 h were stained for the ISG viperin, we observed only very sporadic positive staining, whereas the epithelium exposed to 3’3’-cAIMP was highly positive for viperin (Fig 6C). At later time points after treatment with TLR agonists, more extensive viperin positive staining was observed in the vagina, but with more focal staining patterns in both the epithelium and subepithelial areas (Fig 6D). At 6 h post treatment, significantly elevated levels of *Ifnb* and *Mx1* mRNA were observed in the vaginal tissue after treatment with 3’3’-cAIMP, but not TLR agonists (Fig 6E and 6F). Despite this, the TLR9 agonist induced *Tnfa* expression to an extent comparable to what was observed after STING activation (Fig 6G).

These results demonstrate that STING agonists directly targets epithelial cells, and that vaginal administration induces highly IFN-focused local responses. By contrast, TLR agonists do not directly target epithelial cells, and hence induce vaginal IFN responses with a delayed kinetics, and a stronger associated inflammatory gene expression profile.

## Discussion

Upon infection by a pathogenic microorganism, the body possesses numerous mechanisms to mount defenses that will clear the infection without causing major tissue damage. However, even in individuals with apparently full immune-competence, many infections are not well controlled, but can be cleared by anti-microbial treatments. In immunocompromised individuals, continuous treatment with antimicrobials provides an opportunity for microbes to develop drug resistance, thus hindering microbial control, and increasing the risk of severe disease [6]. Therefore, in recent years there has been increasing interest in not only directing therapy towards the infecting pathogen, but also in developing therapies that stimulate the immune system in a manner targeted towards elimination of the infections [45, 46].

STING is an adaptor protein involved in DNA-activated innate immune signaling, the importance of which we have only just begun to unravel [47, 48]. STING is involved in innate defense against infections with a range of different DNA-containing pathogens, such as viruses [49], intracellular bacteria [50, 51], and protozoa [52], but it is also implicated in autoinflammatory diseases, most notably STING-associated vasculopathy with onset in infancy [53]. Here, we show that it is possible to exploit the former role of STING in antiviral defense by treatment with STING agonists. We found that STING agonists confer protection against genital HSV2 infection in mice when applied systemically or locally. The therapeutic effects were more pronounced in *cGas*^*-/-*^ mice as compared to WT mice, suggesting that endogenous activation of the STING pathway in WT mice did confer some degree of protection. The antiviral action of the CDNs could be mimicked when applying supernatants from CDN-stimulated THP-1 cells (human monocyte-like cell line) onto WT HaCaT cells (human keratinocyte cell line highly permissive for HSV-1), but not IFNAR-deficient HaCaT cells. This suggests that CDNs target a range of cell types and activate antiviral cross-talk between different cell types.

The suggestion of an effect on different cell types led us to test the STING agonists in an *in vivo* model. We confirmed previous findings [51, 54], that CDNs induce a robust type I IFN response, and that they do so in a STING-dependent manner, with much less induction of genes stimulated by the NFκB-pathway. Moreover, we found that a panel of non-endogenous CDNs were superior to 2’3’-cGAMP in stimulating IFN responses *in vivo* while having comparable activity *in vitro*. Furthermore, 2’3’-cGAMP did not induce IFN responses in the brain, whereas the non-endogenous CDN 2’3’-cGAM(PS)_2_ (Rp/Sp) induced type I IFN in all tested tissues. This could indicate an ability of this CDN to better protect neurons from neuroinvasive viruses, although intrathecal type I IFN-synthesis has also been linked to neurotoxicity [55–58]. A mechanistic explanation as to why the non-endogenous CDNs were more potent than the endogenous 2’3’-cGAMP could be that the non-endogenous CDNs are resistant to hydrolysis by phosphodiesterases in the body. ENPP1 has been shown to efficiently degrade 2’3’-cGAMP, and has been found in the plasma as well as on the outside of the cell membrane [28]. Since 2’3’-cGAM(PS)_2_ (Rp/Sp), has been shown to be a competitive inhibitor of ENPP1 [28], treatment with CDN would also potentially amplify the response to endogenously produced 2’3’-cGAMP. The results are consistent with the model shown in S2 Fig. With respect to the current knowledge on STING-directed therapy against disease, DMXAA was shown to have antitumor effects even before its mechanism of action through STING was known [30, 31, 59]. CDNs have also been investigated for anticancer effects which was shown to be mediated by endothelially-produced type I IFN and correlated with the generation of more CD8^+^ T cells [35]. CDNs have been investigated as potential adjuvants on mucosal surfaces as they induced Th1, Th2 and Th17 cells [38, 40, 60]. The induction of type I IFN by CDNs activates dendritic cells [51, 54], thus promoting maturation of CD4^+^ T cells [61, 62]. However, recent papers from the Manel and Poltorak groups [63, 64] suggest that CDNs could impair T cell-based immunity, which is known to be essential for full clearance of many viruses [65, 66]. Therefore, the characterization of STING agonists in therapy should also include the impact on adaptive immunity.

Previous studies have tested the effects of TLR agonists in models for genital herpes. While TLR3 and TLR9 agonists led to efficient protection against disease in mice, a TLR7 agonist was less efficient [67–69]. However, in the guinea pig model for genital herpes, the TLR7 agonist imiquimod potently induced protection. At the mechanistic level, all the tested TLR agonists work in a manner dependent on type I IFN [70], and at least for the TLR9 agonist, the antiviral action was independent of IFNγ [67, 71]. In addition, the TLR agonists potently induce expression of inflammatory cytokines with potential adverse effects for patients. Moreover, there are data suggesting that imiquimod also exerts biological activities through stimulation of the adenosine receptor A_1_-pathway, including upregulation of cystatin A [67, 72]. Despite the promising data on TLR agonists in animal model systems [67–69], and case reports on clinical effects of imiquimod treatment in individual patients [13, 14], imiquimod treatment failed to demonstrate significant effect in a clinical trial on genital herpes [10, 11]. In addition, TLR agonists showed significant inflammatory side effects [10, 11]. The results of our study together with the existing literature suggests that STING and TLR agonists target different cell types and have different modes of action. While, TLR7/9 agonists mainly target plasmacytoid dendritic cells [73, 74], and hence rely on recruitment to the infected tissue, STING agonists act directly on epithelial cells to induce IFN/ISGs. In addition, TLR agonists are stronger inducers of inflammatory cytokines, such as TNFα, than STING agonists are. Collectively, this suggests that local treatment with STING agonists leads to a rapid IFN/ISG response aimed at the epithelial cells, and with limited inflammation. It should also be mentioned that STING agonists potently induce autophagy and different types of programmed cell death, which have been reported to have antiviral activity [75, 76]. Therefore, STING agonists have potential as a novel therapeutic option for treatment of genital herpes, due to their small molecular size and targeted type I IFN-biased response, which in addition induces type III IFN, autophagy, programmed death pathways, and low levels of inflammatory cytokines. The treatment potential includes acyclovir-resistant HSV, which is a problem in immunocompromised patients permanently treated with nucleoside analogs [6]. Finally, future studies should address the clinically important question on the impact on herpesvirus latency and reactivation.

In conclusion, we report that natural and non-natural STING agonists induce expression of type I IFNs and ISGs in human immune and tissue cells, and that this stimulates type I IFN-dependent intercellular cross-talk. This was found to stimulate anti-HSV2 activity both *in vitro* in human cells and *in vivo* in a mouse model for genital herpes. Most notably, mucosal administration of the STING agonist 3’3’-cAIMP evoked a local IFN response, which conferred total protection against genital herpes, even in a highly permissive mouse strain. Such data highlight the potential for immunotherapy in treatment of virus infections and suggest STING-directed therapy to hold a potential that should be further explored.

## Methods and materials

### Animals

Mice were age-matched (6–8 weeks of age) female C57BL/6J (WT), *STING*^*gt/gt*^ (STING-Goldenticket; C57BL/6J-*Tmem173*^*gt*^/J) and *cGAS*^*-/-*^ (B6(C)-*Mb21d1*^*tm1d(EUCOMM)Hmgu*^/J), and all experiments were carried out at Aarhus University.

### Cells

Vero cells were obtained from ATCC (CLL-81TM) and were grown in DMEM with 5% FCS supplemented with L-glutamine and antibiotics. HaCaT cells (ATCC HB-241) were grown in DMEM and THP-1 cells (ATCC TIB-202) in RPMI with 10% FCS and supplemented as described above.

### STING and TLR agonists and delivery

All STING and TLR agonists were supplied by InvivoGen: 5,6,-dimethylxanthenone-4-acetic acid (DMXAA, vadimezan), 2’3’-cyclic guanosine monophosphate-adenosine monophosphate (2’3’-cGAMP), 3’3’-cyclic di-adenosine monophosphate (3’3’-c-di-AMP), the Rp,Sp-isomers of the bisphosphorothioate analog of the mammalian cyclic dinucleotide 2’3’-cGAMP (2’3’-cGAM(PS)_2_ (Rp/Sp)), 3’3’-cyclic adenosine monophosphate-inosine monophosphate [77], imiquimod, ODN1826, and ODN2216. STING agonists were diluted in PBS (with 10% DMSO for DMXAA) and administered by local application or i.p. injections. STING agonists were added to the cell medium. In some experiments, the cells were pretreated with digitonin to permeabilize the cell membrane. In brief, medium was removed and cells were permeabilized with digitonin (5 μg/mL) and buffer (0.2% BSA, 50 mM HEPES, 100 mM KCl, 3 mM MgCl_2_, 0.1 mM DTT, 85 mM sucrose, 1 mM ATP, 0.1 mM GTP, 2.2 mM NaOH) together with the respective drug for 10 minutes. New antibiotics-free medium was then supplied. TLR agonists were diluted in PBS. Mock treatment was performed with PBS in an appropriated volume.

### Virus infections

One preparation of HSV2 333 strain was used for all experiment and produced as previously described [41]. Cells were infected with an MOI of 0.1. The mice were pretreated with a subcutaneous (s.c.) injection of 2 mg Depo-Provera (medroxyprogesterone acetate; Pfizer). Five days later, the mice were anesthetized and inoculated intravaginally with 20 μL HSV2 (6.7×10^4^ p.f.u.) suspended in IMDM. The mice were then placed on their backs for 10 min. Vaginal washes were collected 48 h post infection (p.i.) by washing with 40 μL of IMDM and dilution to a final volume of 200 μL. In the survival experiments, infected mice were weighed and monitored for disease symptoms daily and euthanized when they reached humane endpoints: weight loss >10%, severe inflammation with ulceration in the genitoanal region, paresis of the hind limbs, or hunchback with antisocial behavior and facial expressions of pain [78].

### Whole-animal transcardial PBS perfusion

The mouse was anesthetized and the right heart atrium was punctured. A feeding needle was used to infuse PBS, 10 mL/min, through the left ventricle, clearing the circulation of blood. Successful perfusion was confirmed by the color change of the liver from deep red-brown to pale brown.

### IFN assay

Sera from mice were analyzed for IFNμ and -β by Bioluminescent LumiKine Xpress ELISA (luex-mifnb and luex-mifna; InvivoGen) as described by the manufacturer. Human type I IFN was assessed by HEK-Blue™ IFN-α/β reporter cells by the manufacturer guide lines (InvivoGen).

### Immunohistochemistry of vaginal tissue

Vaginal tissues were fixated in 10% formalin, embedded in paraffin, and cut at 4 μm. The tissues sections submitted to antigen retrieval in a citrate buffer and blocked in 5% BSA. The sections were incubated overnight with primary antibody for HSV2 (B0116; Dako) and viperin (MABF106; EMD Millipore), and the stain was visualized with appropriate secondary antibodies from Molecular Probes. DAPI was used to stain cell nuclei.

### End-point dilution assay for HSV2 quantification

37,500 Vero cells were seeded into each well of a 96-well plate. The next day, sample was added in 8 replicates and serially diluted. The result was read at 48 h p.i., using light microscopy, and the 50% tissue culture infective dose (TCID50) was calculated, using the Reed-Muench method [79]. Data represented as scatter plots with the geometric mean ± SD.

### Western blot and antibodies

Protein was isolated with RIPA buffer supplemented with protease inhibitors, and the protein concentration was measured with a Bradford protein assay. The samples were run on a reduced gel, and the membrane was blocked in either 2.5% BSA (for phosphorylated proteins) or 5% skim milk. The following primary antibodies were used: anti-vinculin (V9131; Sigma), anti-viperin (MABF106; EMD Millipore), anti-STING (AF6516; R&D), anti-VP5 (Ab6508; Abcam), anti-pSTAT1 (7649S; Cell Signaling), ISG15 (2743S; Cell Signaling). Appropriate secondary antibodies from Jackson ImmunoResearch were used. The blots were visualized with an ImageQuant LAS 4000 Mini Luminescent Image Analyzer (GE Healthcare).

### RT-qPCR

RNA from non-CNS tissues was isolated with the RNeasy Mini kit with on-column DNase digestion (Qiagen). RNA from CNS tissues was isolated with TRIzol (Ambion) and digested with DNase I (InvitroGen). Gene expression was determined by reverse transcriptase quantitative PCR (RT-qPCR), using the SYBR Green (Agilent Technologies) and TaqMan (Applied Biosystems) systems. 50 ng of RNA was used for each reaction. Expression levels were quantified relative to the expression of GAPDH, using the $2^{-\Delta \Delta C_{T}}$ method [80], and normalized to the control group as a fold change. Data are represented as scatter plots with the mean ± SD of biological replicates. The following TaqMan primers were used: *mIfnb* (Mm00439552_s1; Applied Biosystems), *mMx1* (Mm00487796_m1; Applied Biosystems). The following SYBR Green primers were used: *mGapdh* (FW: 5’-CAA TGT GTC CGT CGT GGA-3’; RW: 5’-GAT GCC TGC TTC ACC ACC-3’), *mA20* (FW: 5’-TGC AAT GAA GTG CAG GAG TC-3’; RW: 5’-TGG GCT CTG CTG TAG TCC TT-3’), *mIl6* (FW: 5’-GAA AAT CTG CTC TGG TCT TCT GG; RW: 5’-TTT TCTG ACC ACA GTG AGG AAT G), *mTnfa* (FW 5’-CAC AGC CTT CCT CAC AGA GC; RW: 5’-GGA GGC AAC AAG GTA GAG AGG).

### NF-κB reporter gene assay

THP1-Dual Cells (thpd-nfis) reporter cell line was obtained from InvivoGen and enable detection of the ISG54 and the NFκB pathways. Briefly, this cell line expressed a SEAP reporter gene under control of a promoter that is responsive for the NF-κB or AP-1 pathways and a Lucia reporter gene under the control of a promoter that comprises five IFN-stimulated response elements (ISREs) fused to a minimal promoter of the human ISG54 gene. 24h stimulation of this cell line with CDN analogs subsequently induces production of Lucia and SEAP, which are measured using Quanti-Luc and Quanti-Blue respectively.

### Biostatistics

All statistical analyses were performed with GraphPad Prism version 7.03 for Windows, GraphPad Software, La Jolla California USA, www.graphpad.com. Statistical significance of p<0.05 was marked with an asterisk (*). For details about the statistical procedures, please refer to the respective figure legends. In general, all analyses were initially sought to be carried out with parametric procedures (one-way ANOVA, two-way ANOVA) and adequate post-hoc tests for multiple comparisons (Šidák correction, Dunnett’s test). Virus titers were log_10_-transformed prior to analysis [81]. When the underlying assumptions of the parametric test were not met, e.g. the data were not normally distributed or the SDs were significantly different as tested by Prism, we used non-parametric tests (Mann-Whitney-Wilcoxon U test, Kruskal-Wallis test) and adequate tests for multiple comparisons (Dunn’s test). Comparison of overall survival was done with the log-rank test and the post-hoc Holm-Bonferroni correction.

### Ethics statement

Animal studies were reviewed and approved by Dyreforsøgstilsynet under the Danish Ministry for Veterinary and Food Administration (permission#: 2015-15-0201-00686). The study was carried out in strict accordance with the recommendations in the Guide for the Care and Use of Laboratory Animals, EEC Council Directive 2010/63/EU.

## Supporting information

S1 Fig STING agonists induce an IFN response, but not an NF-κB response. (**A**) Mice were injected with 500μg DMXAA i.p. and the expression of *Ifnb*, *Mx1* and *A20* were measured with RT-qPCR. n = 5. * = p<0,05 compared to NT. (**B**) Equimolar (1.687x10^-7^ mol) doses of 2’3’-cGAMP(PS)_2_ or 3’3’-cAIMP were administered to mice i.p. Samples were collected 6 hours later. Gene expression for *Tnfa*, *Il6* and *A20* on tissues samples from vagina and spleen normalized to *Gapdh*. n = 3–5. (**C**) Wildtype, *cGas*^-/-^ and *Sting*^*gt*/*gt*^ mice were treated with 2’3’-cGAMP(PS)_2_ for 6 hours and the expression of *Ifnb*, *Mx1* and *A20* in the spleen were measured and normalized to *Gapdh*. n = 3–5. * = p<0,05 compared to mock. (**D**) THP1-Dual^TM^ cells were stimulated with various doses of 2’3’-cGAM(PS)_2_ (Rp/Sp), 2’3’-cGAMP or 3’3’-cAIMP for 24 hours and NFκB activity was measured by reading the presence of alkaline phosphatase secreted in the supernatant upon addition of Quanti-Blue^tm^ substrate (absorbance at λ = 630nm). Results presented are from 5 independent experiments. Statistics, (**A**, **B**) Kruskal-Wallis test with Dunn’s multiple comparisons test. (**C**) One-way ANOVA with Šidák’s multiple comparisons test.
(TIF)

S2 Fig Model for the mode of action of therapeutic treatment with STING agonists in genital herpes. (A) In the absence of treatment, epithelial cells and recruited leukocytes induce a weak type IFN response during HSV2 infection, exerting moderate antiviral activity that does not confer protection from disease. (B) Systemic treatment with STING agonists leads to rapid and strong induction of systemic IFN responses, which is most pronounced for the non-natural CDNs, possibly due to inefficient degradation by e.g. ENPP1. The IFN response induced in the vagina confers protection against HSV. (C) Local treatment with CDNs in the vagina induces a strong IFN response in epithelial cells, with only limited systemic effects. This response confers complete protection against genital HSV2 disease.
(TIF)
