# Supplementary figures and images for: STING agonists enable antiviral cross-talk between human cells and confer protection against genital herpes in mice
Source: PLoS Pathog. 2018 Apr 2;14(4):e1006976. doi: 10.1371/journal.ppat.1006976 (PMC5897032; doi:10.1371/journal.ppat.1006976)

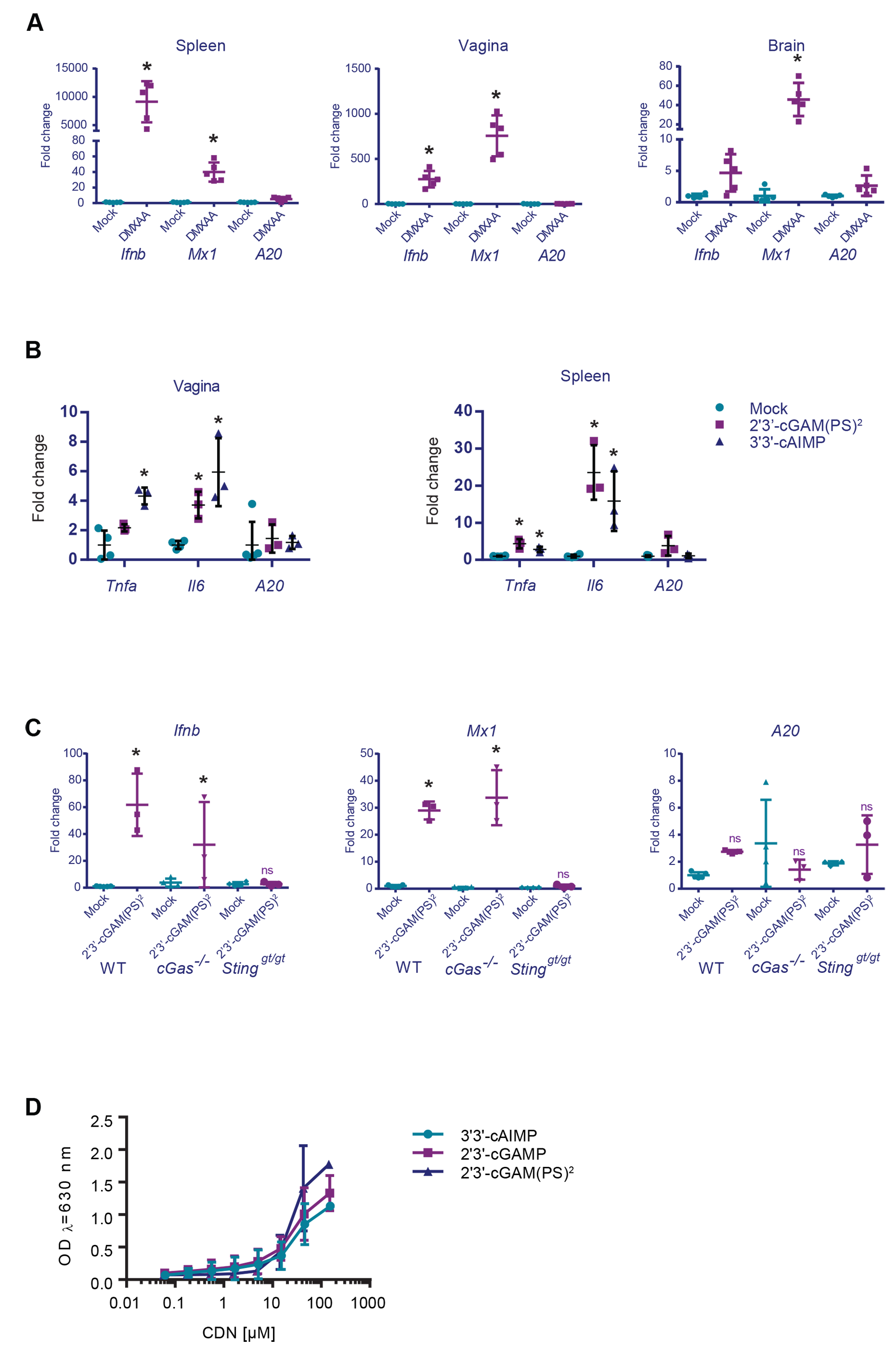

Supplement: S1 Fig — (A) Mice were injected with 500μg DMXAA i.p. and the expression of Ifnb, Mx1 and A20 were measured with RT-qPCR. n = 5. * = p<0,05 compared to NT. (B) Equimolar (1.687x10^-7^ mol) doses of 2’3’-cGAMP(PS)2 or 3’3’-cAIMP were administered to mice i.p. Samples were collected 6 hours later. Gene expression for Tnfa, Il6 and A20 on tissues samples from vagina and spleen normalized to Gapdh. n = 3–5. (C) Wildtype, cGas-/- and Stinggt/gt mice were treated with 2’3’-cGAMP(PS)2 for 6 hours and the expression of Ifnb, Mx1 and A20 in the spleen were measured and normalized to Gapdh. n = 3–5. * = p<0,05 compared to mock. (D) THP1-DualTM cells were stimulated with various doses of 2’3’-cGAM(PS)2 (Rp/Sp), 2’3’-cGAMP or 3’3’-cAIMP for 24 hours and NFκB activity was measured by reading the presence of alkaline phosphatase secreted in the supernatant upon addition of Quanti-Bluetm substrate (absorbance at λ = 630nm). Results presented are from 5 independent experiments. Statistics, (A, B) Kruskal-Wallis test with Dunn’s multiple comparisons test. (C) One-way ANOVA with Šidák’s multiple comparisons test. (TIF) [file ppat.1006976.s001.tif]

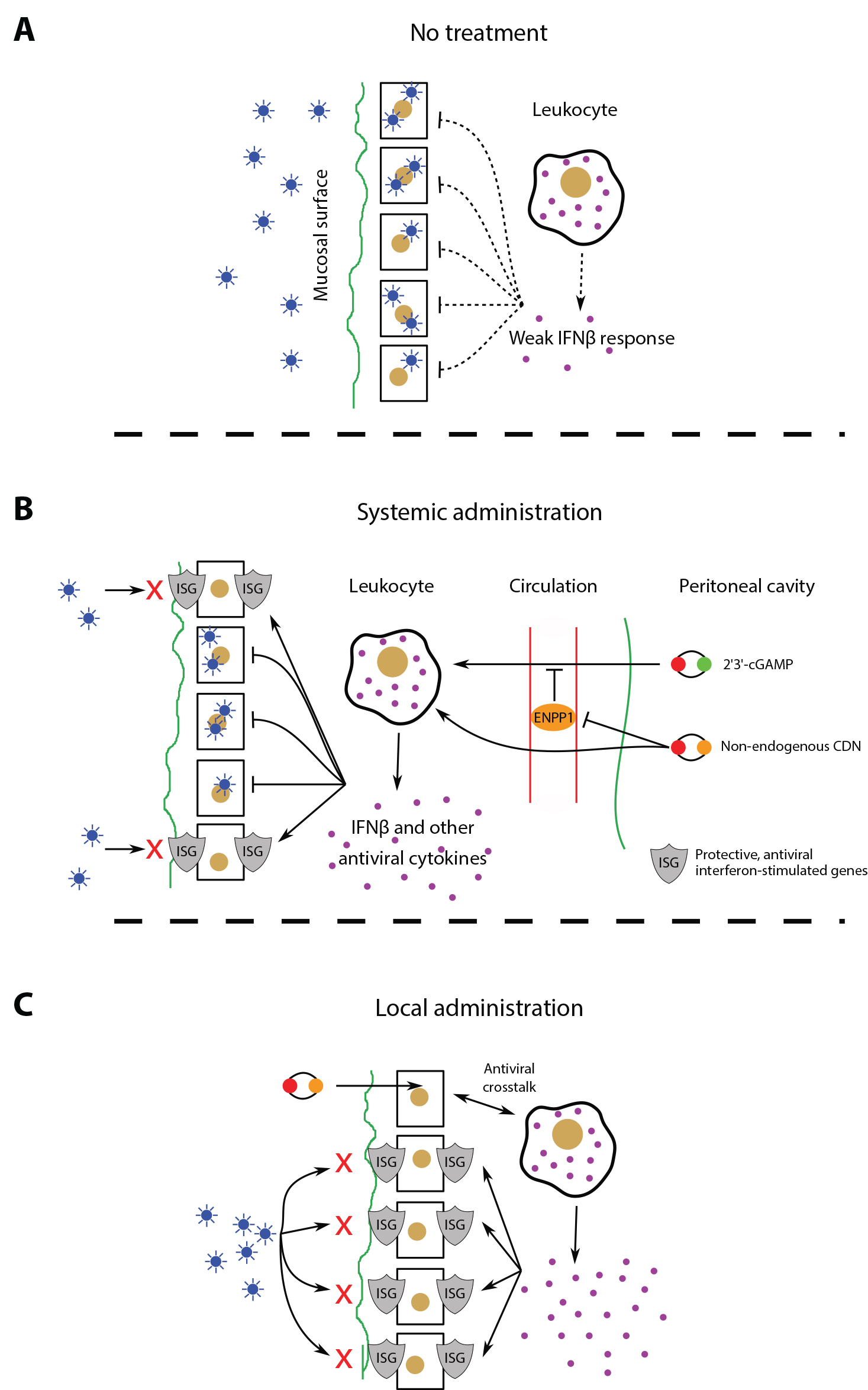

Supplement: S2 Fig — (A) In the absence of treatment, epithelial cells and recruited leukocytes induce a weak type IFN response during HSV2 infection, exerting moderate antiviral activity that does not confer protection from disease. (B) Systemic treatment with STING agonists leads to rapid and strong induction of systemic IFN responses, which is most pronounced for the non-natural CDNs, possibly due to inefficient degradation by e.g. ENPP1. The IFN response induced in the vagina confers protection against HSV. (C) Local treatment with CDNs in the vagina induces a strong IFN response in epithelial cells, with only limited systemic effects. This response confers complete protection against genital HSV2 disease. (TIF) [file ppat.1006976.s002.tif]
